# Dispersal of an ancient retroposon in the TP53 promoter of Bovidae: phylogeny, novel mechanisms, and potential implications for cow milk persistency

**DOI:** 10.1186/s12864-015-1235-8

**Published:** 2015-02-05

**Authors:** Yaron Dekel, Yossy Machluf, Shifra Ben-Dor, Oren Yifa, Aviad Stoler, Izhar Ben-Shlomo, Dani Bercovich

**Affiliations:** MIGAL – Galilee Research Institute, Kiryat Shmona, Israel; Weizmann Institute of Science, Rehovot, Israel; Faculty of Medicine in the Galilee, Bar Ilan University & Baruch Padeh Medical Center, Poria, & Zefat Academic College, Safed, Israel; Tel Hai College, Upper Galilee, Israel; Golan Research Institute, Katzrin, Israel

**Keywords:** TP53 Promoter, Bov-A2, Molecular Phylogeny, Milk Persistency, Palindrome

## Abstract

**Background:**

In recent years, the perception of transposable genetic elements has changed from “junk DNA” to a focus of interest when appearing near or inside genes. Bov-A2 is a short interspersed nuclear element (SINE) that was first found in Bovidae and later in other ruminants. This retroposon is mostly used as a marker for phylogenetic analysis.

**Results:**

We describe insertions of Bov-A2 in the promoter region of TP53, a key tumor suppressor gene that is indispensable for diverse developmental processes, in Antilopinae and Tragelaphini (belonging to the Bovinae subfamily). In Tragelaphini two Bov-A2 elements were inserted sequentially, whereas in 5 tribes of Antilopinae only one Bov-A2 element was inserted, in a different site and reverse orientation. The entrance site in both cases employed short palindromes that can form hairpin secondary structures. Interestingly, mutations that create or disrupt base pairing in the palindrome sequence dictated the presence or absence of Bov-A2, such as in the domestic cow and buffalo, which lack Bov-A2. Transcription factor binding site analysis revealed unique binding sites for STAT3 and NFκB within the Bov-A2 sequence, which together with TP53 itself are known to play a crucial role in mammary involution.

**Conclusions:**

This report demonstrates how short palindromes serve as hot spots for Bov-A2 retroposon insertion into the mammalian genome. The strict correlation between point mutation in the palindromes and the presence/absence of Bov-A2 retroposon insertions, questions the use of singular insertion events as valid phylogenetic markers inside families. Bov-A2 insertion into the TP53 promoter in Antilopinae and Tragelaphini may not only provide a genetic network that regulates mammary involution, but can also answer the need for rapid mammary involution in Savanna antelopes after weaning, partially in response to predation stress. The absence of Bov-A2 in domestic bovids may constitute the molecular background for greater lactation persistency.

**Electronic supplementary material:**

The online version of this article (doi:10.1186/s12864-015-1235-8) contains supplementary material, which is available to authorized users.

## Background

Transposable genetic elements have been known for decades since the pioneering work of Barbara McClintock [1]. However, only in recent years, with the aid of novel massive parallel sequencing technologies and computational power, can these elements be more easily identified, classified and studied [2,3]. The largest class of sequences in mammalian genomes is interspersed repetitive elements that can constitute, in some cases, more than 50% of the genome [4,5]. Retrotransposons and retrotransposon-like elements (RTE), such as retroposons, are the most common repeats. These elements replicate in a manner similar to retroviruses, via DNA transcription followed by reverse transcription of their RNA intermediate and finally DNA integration into the genome [6]. The abundance of ruminant-specific repeats, such as BovB, Bov-tA, Bov-A2 and ART2A, is negatively correlated with gene density and GC content [7].

Most retroposons and RTEs were considered non-functional and thus regarded as “junk DNA”. At present, after gaining more information and understanding of control mechanisms, there are increasing indications that retroposons can serve different functions in the evolutionary process of exaptation [8]. Promoters, enhancers, silencers and chromatin modifiers are only part of the growing list of control mechanisms that have been reported to involve these elements [4]. Genomic repeats are particularly abundant in promoters and *cis*-regulatory elements, in particular transposable elements, which may serve as a rich source of material for the assembly of and tinkering with eukaryotic gene regulatory systems [9,10]. They may also be involved in post-transcriptional regulation of gene expression (for example, as small RNA and miRNA producers as well as controlling elements of RNA stability) and possibly even in translation regulation [11]. A growing body of evidence now indicates a close association of transposable elements with non-coding RNAs (ncRNA), which in turn may participate in a wide-range of regulatory functions, as well as be linked to disease [12]. By re-wiring regulatory networks, the mobilome (including RTEs) can facilitate the evolution of complex and novel physiological processes involving gene expression on a global scale. An intriguing example for this is the evolution of pregnancy [13].

Bov-A2 is a retroposon that was first discovered in bovid species and therefore was named after the family Bovidae [14]. It was later shown to be widely distributed among other ruminant genomes [15]. The element is composed of two Bov-A monomers which are joined together by a linker sequence, and ends with a [AGC]n repeat [16,17]. Bov-A2 is usually categorized as a member of the short interspersed nuclear element (SINE) family [11,14,18]. Notably, Onami and colleagues proposed that due to the absence of promoter sequences for RNA polymerase III and the homology of its two units to the Bov-B long interspersed nuclear element (LINE), Bov-A2 does not belong to a SINE family but rather is a kind of retroposon that is transcribed by RNA polymerase II [16]. Events of SINE excision from its location in the genome are extremely rare, thus making it a suitable genetic marker for taxonomy [19]. Several studies on the distribution of Bov-A2 in the ruminant genome have been conducted. Bov-A2, along with other members of the Bov-A SINEs and Bov-B LINEs, constitutes an important marker in deciphering the evolution and phylogeny of ruminants [11,19].

Bovidae, a family belonging to ungulate ruminants, consists of two subfamilies, Bovinae and Antilopinae. According to common classifications [20,21], these subfamilies are, respectively, of Eurasian and African origin [22]. In total, the family comprises almost 140 extant species and more than 300 fossil species. However, the phylogenetic relationships and taxonomy of Bovidae are still controversial [23,24]. Bovidae include the economically most important species to mankind - the cow (*Bos taurus*), which was among the first livestock animals to be domesticated around 9000 B.C. [25,26], close to the time of the domestication of goats and sheep [27,28]. Recently, it was claimed that the former followed the later [29], though the order is still under genomic [30] and archaeological [31] research and debate. Due to its importance as the main milk and meat source in the world, the cow was the first livestock mammal whose genome was fully sequenced [32,33], and efforts are devoted to support annotation of the bovine genome [34].

Stemming from a search of differences in key regulatory genes, which might serve as the genetic background for domestication or adaptation to ecological niches, we analyzed the P1 promoter sequence of TP53, among others. Here, we report on the localization of Bov-A2 in the promoter region of the tumor suppressor gene TP53 in Bovidae. This SINE was inserted in reverse orientation into the TP53 P1 promoter of tribes belonging to Antilopinae, clearly defining this subfamily from Bovinae. The assumed mechanism of insertion was found to involve a palindromic conserved sequence in all Antilopinae tribes that we sequenced. Surprisingly, in Tragelaphini, a tribe belonging to the subfamily Bovinae, two Bov-A2 elements were inserted sequentially, in the original orientation and at a different site located 39 bp downstream to the Antilopinae insertion site. This probably occurred due to a second palindromic sequence that carries a mutation specific to spiral-horn antelopes and is absent from the genomes of the rest of the members of the family. In domestic cattle (cow and zebu, of European and Asian origin, respectively), Bov-A2 is absent from the TP53 P1 promoter region, most likely due to a point mutation in the first palindromic entrance site. Computational analysis of the ruminants’ TP53 P1 promoter revealed putative RTE-specific transcription factor binding sites (TFBS) which cause physiological and evolutionary repercussions. We detail the findings and discuss the possible implications to the evolution of and phenotypic differences within the Bovidae.

## Results

### Analysis of the TP53 P1 promoter of Bovidae

**Figure 1:**
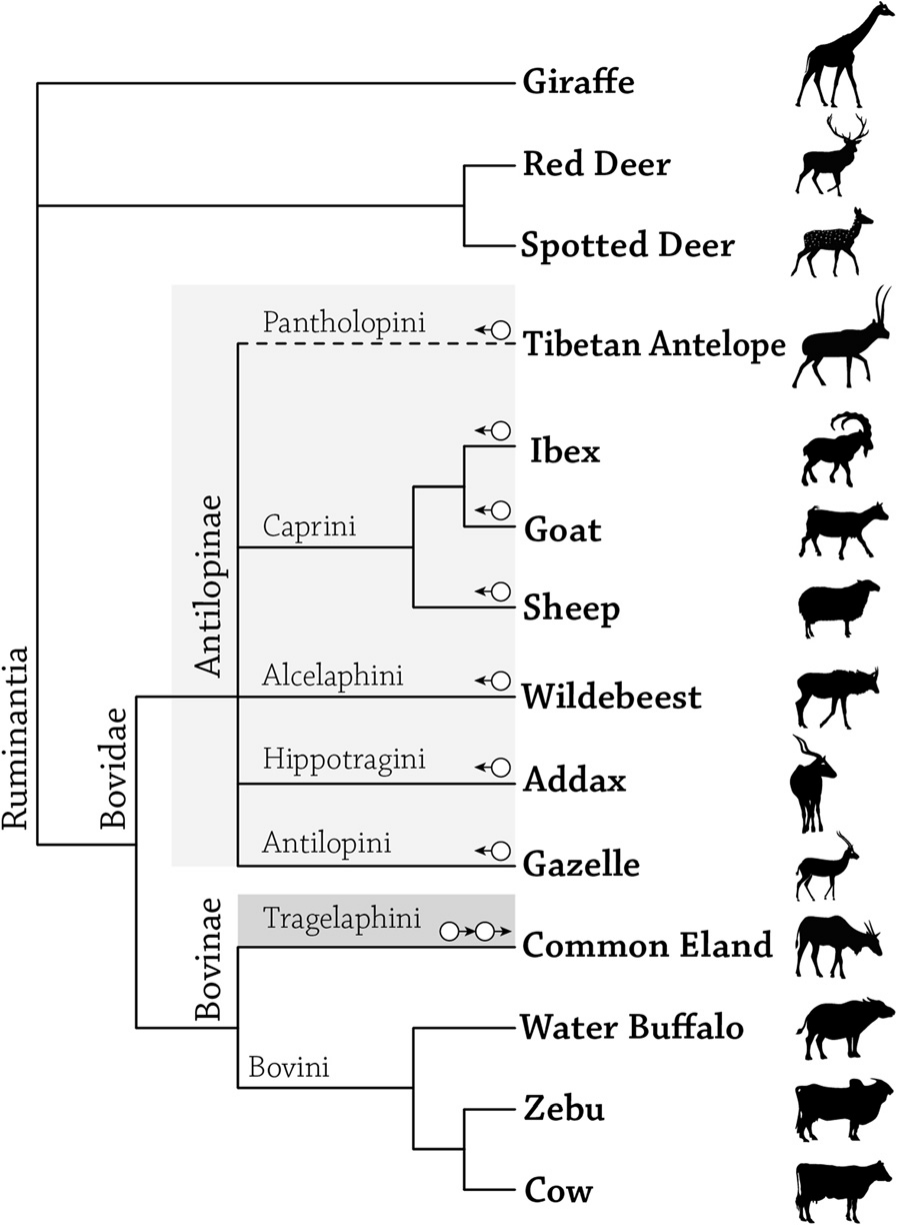
**A schematic phylogenetic tree of 11 Bovid species representing Bovidae’s two subfamilies and seven tribes.** Three species from two other ruminant families were used as outgroups (giraffe and deers). Each circle represents an insertion of the Bov-A2 retroposon element into the TP53 P1 promoter. The arrow indicates its orientation. A continuous line indicates sequences which were determined experimentally, whereas a dashed line indicates sequences obtained by bioinformatics means.

The P1 promoter region of TP53 in the *Bos taurus* (domestic cow) genome was determined by transcript and EST analysis, and confirmed by alignment to the known TP53 P1 promoter in humans. The P1 promoter of TP53 is approximately 1.5 kb upstream to the TP53 transcription start site (TSS), which is composed of a 1088 nucleotide-long non-coding region and most of the WRAP53 first exon. The sequence of the P1 promoter was determined experimentally by PCR amplification, gel electrophoresis and subsequent sequencing. Sequence analysis revealed a product 272 bp longer than the one found in the cow in all Antilopinae tribes analyzed in this study, including: ibex (*Capra nubiana*), goat (*Capra aegagrus hircus*), sheep (*Ovis aries*), wildebeest (*Connochaetes taurinus)*, addax (*Addax nasomaculatus*) and gazelle (*Gazelle gazelle*) (Figure 1). The additional 272 bp segment, which is located approximately 500 bp upstream of the TP53 TSS, is not indicated in the published genome of sheep, as opposed to the goat genome (data not shown). Bioinformatics analysis identified the same additional 272 bp segment in the same location within the TP53 P1 promoter of Tibetan antelope (*Pantholops hodgsonii*), a species belonging to the Pantholopini tribe (Figure 1). In order to further investigate this evolutionary conservation among tribes belonging to the Antilopinae subfamily, we looked into the TP53 P1 promoter of three additional species of the Bovinae subfamily: one belonging to the Tragelaphini tribe (the common eland from spiral horned antelopes - *Taurotragus oryx*) and two belonging to the Bovini tribe (water buffalo - *Bubalus bubalis*, and zebu - *Bos indicus*). In the zebu and buffalo, similarly to the cow, the extra 272 bp long segment was not found, whereas in the common eland a segment of 522 bp was found in a location 39 bp downstream to the Antilopinae entrance site (Figure 1). It appears that the insertion into the TP53 P1 promoter, in terms of size and location, resulted from two independent and unrelated insertion events which occurred after the split between the two subfamilies (approximately 23 million years ago [22]): one ancient and common to all seven (analyzed) Antilopinae species, and one unique to the tribe Tragelaphini. Examination of the same promoter region in the outgroups in this study, two close families of bovidae, Cervidae (represented by red deer - *Cervus elaphus*, and spotted deer - *Axis axis*) and Girraffidae (represented by giraffe - *Giraffa camelopardalis*), revealed no insertion, similar to the condition in Bovini (Figure 1).

### Characterization of the Bov-A2 element in the TP53 P1 promoter of Bovidae

**Figure 2:**
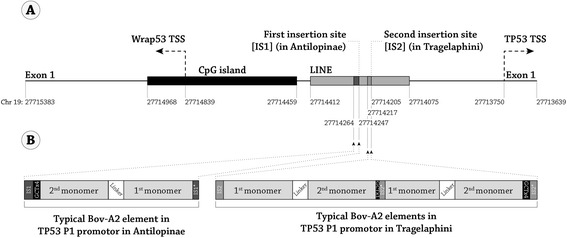
**The insertion of Bov-A2 retroposon element into the TP53 P1 promoter. (A)** A schematic representation of the genomic region of the TP53 P1 promoter in cow. The genomic cow chromosome 19 coordinates are indicated, based on the Baylor Btau_4.6.1/bosTau7 October 2011 assembly available in the UCSC genome browser (http://genome.ucsc.edu/cgi-bin/hgBlat?command=start). **(B)** The location, orientation and structure of the Bov-A2 retroposon element in the TP53 P1 promoter of Antilopinae (left) and Tragelaphini (right). Abbreviations: TSS- Transcription start site, IS1- Insertion site 1, IS2- insertion site 2.

The additional 272 bp segment in Antilopinae is a SINE insertion that was previously designated as Bov-A2 [14]. The insertion site of Bov-A2, though different in Antilopinae and Tragelaphini, was within a LINE sequence in the TP53 P1 promoter, approximately 500 bp and 460 bp upstream to the TSS, respectively (Figure 2A). In all 8 Bovidae species, representing 6 tribes, that were experimentally and computationally explored here (Tibetan antelope was explored only bioinformatically), the conserved Bov-A2 elements were clearly defined. This retroposon element consists of a two Bov-A monomers connected by a 27 bp linker, and ends with a repetitive terminator sequence [AGC]X_4_. The two Bov-A monomers can be distinguished by polymorphisms at specific loci, termed diagnostic sites, as well as different lengths (the 1^st^ and 2^nd^ monomers are 116 bp and 117 bp long, respectively) [16]. Based on the Bov-A2 components and differences between the Bov-A monomers, one can determine that the Bov-A2 element entered once into the TP53 P1 promoter of the Antilopinae species (sheep, goat, ibex, addax, gazelle, wildebeest, and Tibetan antelope), in the reverse orientation, whereas two Bov-A2’s (rather than Bov-A4) adjacent to each other entered the TP53 P1 promoter of Tragelaphini (common eland) in the forward orientation (Figure 2B).

**Figure 3:**
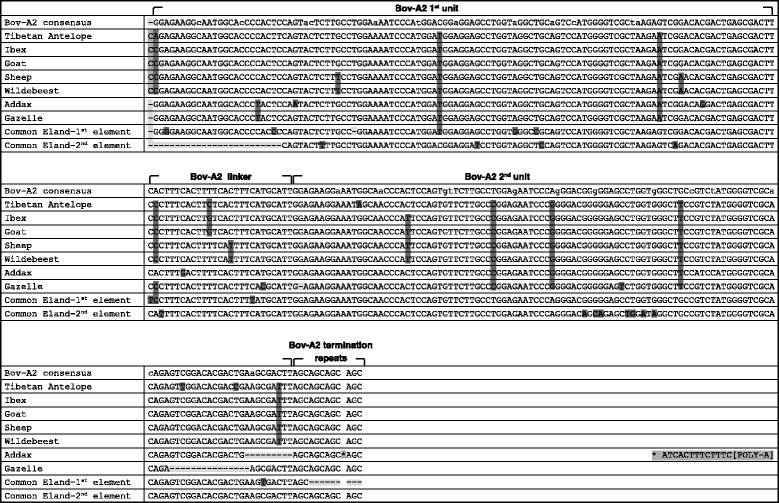
**Multiple alignment of Bov-A2 sequences found in TP53 P1 promoter.** The Bov-A2 consensus sequence is based on literature survey (Onami et al. [16]), and its components’ boundaries are marked. All sequences were determined experimentally, except the Tibetan antelope which was discovered by computational means. Mutations are highlighted: deletion (−) in light gray, nucleotide change in dark gray, and insertion in intermediate gray (a unique insertion in addax in marked by asterisk, and the sequence is depicted at the end of the line). Reverse complement sequences are shown for these species in which the Bov-A2 was inserted in reverse orientation (antelope, ibex, goat, sheep, wildebeest, gazelle).

In comparison with published data [16], some point mutations were found in the first and second Bov-A monomers, the linker and in one instance even in the terminator sequence. The spread of the mutations in the different species and tribes, and their precise locations, are summarized in Figure 3. All of the mutations that are seen in the goat are also found in its wild relative the ibex. In gazelles, the same Bov-A2 mutations also appear, with one exception in the 2^nd^ monomer. In addition, there are 3 species-specific mutations, all in the 2^nd^ monomer. The wildebeest shares all its Bov-A2 mutations with the sheep, although they are from different tribes. As sheep, goat and ibex belong to the same tribe, it was unexpected that the sheep sequence would be more similar to the wildebeest sequence. Multiple sequence alignment of the full TP53 P1 promoter revealed that this finding is indeed valid, since in the whole promoter region the sheep sequence is more similar to the wildebeest (data not shown). The common eland, a member of the Bovinae subfamily, has unique mutations, mainly within the second downstream Bov-A2 retroposon element (closer to the TP53 TSS), that are not seen in its relatives from Antilopinae. This observation strengthens the notion of an independent Bov-A2 insertion event at the TP53 promoter of Bovidae (Figure 3).

### Palindromes act as recognition sequences for the Bov-A2 insertion

**Figure 4:**
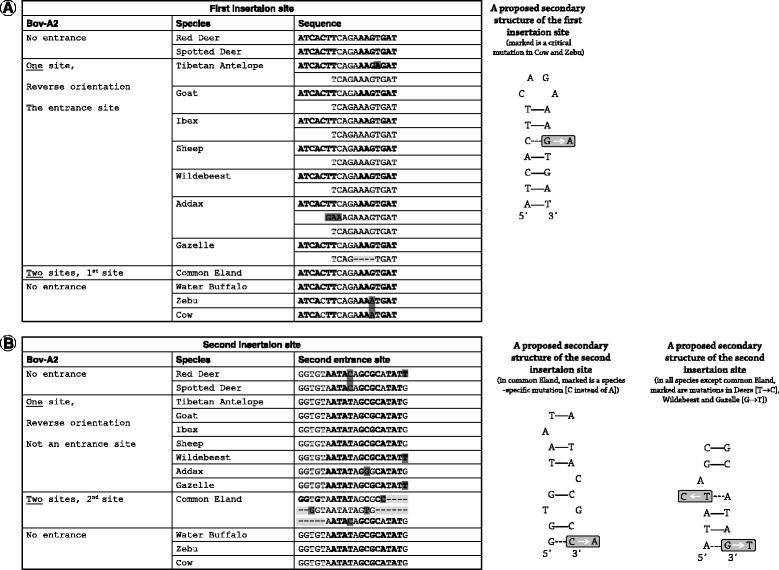
**Multiple alignment (left) and proposed secondary structure (right) of the first (A) and second (B) Bov-A2 insertion sites in TP53 P1 promoter.** Mutations are highlighted: deletion (−) in light gray, and nucleotide change in dark gray. Nucleotides in positions that pair in the secondary structure are marked in bold.

In all of the studied species belonging to Antilopinae, there was one insertion event of the Bov-A2 element in the exact same location and yet in reverse orientation to the one described in the literature. The element resides between two similar sequences, TCAGAAAGTGAT (Figure 4A, left panel). The sequence at the 3′ end is probably a result of non-equal homologous recombination during the insertion into the genome [35,36]. When looking broadly at the sequences of the insertion site, an 18 bp palindrome is detected (Figure 4A, right panel) and its sequence is fully conserved in 6 out of 7 species of Antilopinae. This palindrome will be designated here as PALI1. In the domestic cow (both European and Indian subspecies), a transversion mutation, G to A, at the 14^th^ position of PALI1 results in a mismatch in base pairing (Figure 4) which in turn can impede the ability of this sequence to form three dimensional hairpin structures due to energetic constraints (Additional file 1: Figure S1, free energies [37] and DNA structures based on *mfold* Web Server [38]). Surprisingly, another member of the Bovini tribe, the water buffalo, which lacks a Bov-A2 sequence in the TP53 P1 promoter, has an intact PALI1 sequence and so its sequence is similar to its far relatives in Bovidae and dissimilar from its very close relative the cow. In the common eland, PALI1 is not mutated, and yet Bov-A2s were inserted 39 bp downstream from PALI1, at a different palindromic sequence, designated here as PALI2 (Figures 2A and 4B). In this species (Figure 4B, left panel), PALI2 has one extra nucleotide match due to a species-specific transversion point mutation, A to C, and it lacks the last 4 nucleotides, both resulting in a different base-pairing and a more stable palindrome (Figure 4B, right panel and Additional file 1: Figure S1). The Bov-A2 elements were probably inserted here in two sequential events. In the first event, the insertion occurred following the PALI2 mutated sequence (or perhaps the 4 missing nucleotides are the result of this event). In the second event, the insertion occurred following the newly formed PALI2 sequence that appeared as a consequence of the first event. In both cases, the non-equal homologous recombination resulted in the formation of an additional flanking truncated PALI2 sequence. In the common eland, the existence of a truncated PALI2 sequence (the second site) further supports the notion of two adjacent Bov-A2 elements rather than a single Bov-A4 element. It is interesting to note that in the case of the single Antilopinae insertion event, the truncated repeat of PALI1 is truncated only in its 5′ side. In the case of the common eland, PALI2 is truncated at both the 5′ and the 3′ sides.

There is evidence for a second SINE insertion event in the addax. A unique polyA (more than 10 nucleotides long) is followed by a partial PALI1 sequence, all located between the third and last AGC triplet in the terminator of the first inserted Bov-A2 (Figure 3).

### Transcription factor binding site analysis of the TP53 P1 promoter in Bovidae

**Figure 5:**
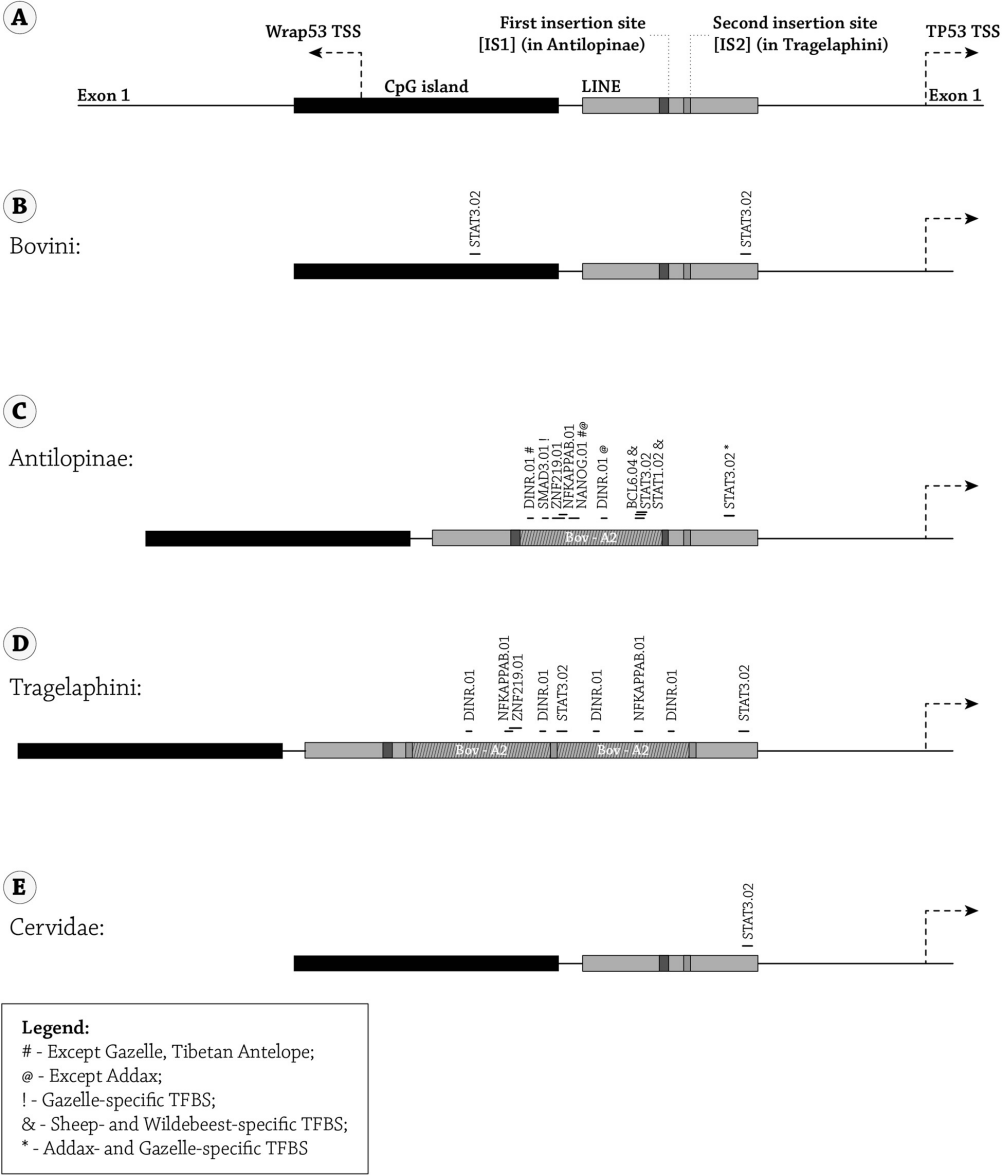
**Transcription factor binding site (TFBS) analysis was performed using the Genomatix Genome Analyzer (GGA) program** **(** **https://www.genomatix.de/solutions/genomatix-genome-analyzer.html** **). (A)** A schematic representation of the of the TP53 P1 promoter genomic region in cow, including the full wrap53 exon 1 until the full TP53 exon 1. **(B-E)** The actual sequence that was analyzed in **(B)** Bovini, **(C)** Antilopinae, **(D)** Tragelaphini, and **(E)** Cervidae included the full CpG island until 50 bp in TP53 exon 1. The location and boundaries of each Bov-A2 element are indicated. The position and size of the unique TFBS are indicated. Abbreviations: TSS- Transcription start site, IS1- Insertion site 1, IS2- insertion site 2.

Transcription factor binding site (TFBS) analysis was performed using the Genomatix Genome Analyzer (GGA) MatInspector program [39]. To this end, the TP53 P1 promoter was analyzed, including the full CpG island (starting within the WRAP53 first exon) until 50 nucleotides downstream of the TP53 TSS (Figure 2), covering at least 1270 bp in the case of SINE-less animals. Raw data for this analysis can be found in Additional file 2: Figure S2. The analysis focused on TFBS sequences that were newly brought into the promoter region by Bov-A2. The Antilopinae Bov-A2 element contains some unique TFBSs, such as DINR (twice), ZNF219, NFκB, NANOG, BCL6 and STAT1, that do not appear outside the Bov-A2 element in the remaining TP53 P1 promoter of Antilopinae or of other Bovini and Cervidae members (Figure 5). The first three TFBSs (DINR, ZNF219 and NFκB) also appear in the Bov-A2 element of Tragelaphini, whereas the last two TFBSs (BCL6 and STAT1) are unique to the sheep and wildebeest. STAT3 appears in most Antilopinae members only in the Bov-A2 element. Only in the addax and gazelle can an additional and conserved STAT3 TFBS be found near the TP53 TSS, as appears also in the Cervidae and other Bovids (Figure 5). Smad3 appears only in the gazelle Bov-A2 and not in any other species in the promoter region screened here. In terms of TFBS which are enriched in the Bov-A2 element, TAL1 appears twice in the Bov-A2 single insertion and 3 times in the Tragelaphini double insertion, and only once in the distal CpG island of all the animals sequenced here.

## Discussion

A significant portion of mammalian genomes consists of repetitive DNA sequence fragments, such as retroposons in the forms of either short or long interspersed elements (SINEs and LINEs, respectively). Because of the clear presence of an element and the low likelihood of exact parallel insertions or deletions, the retroposon presence or absence is considered to be exceptionally effective for detecting ancestral lineages [40]. In the case of bovids, the retroposon Bov-A2, a SINE which is widely distributed in ruminants’ genomes, is detected and used as a genetic marker for evolutionary classification. It was previously shown that very few insertion events, sometimes even a single event, can be used for classification in this important family in which classification is still under debate [19]. Additionally, in different organisms different classes of retro-elements show diverse integration site preferences into gene-rich regions [41,42]. The insertion event of a Bov-A2 element in the TP53 P1 promoter of Bovidae described here questions this general assumption. As we report, at the same exact location in the same family, a point mutation dictates the absence of Bov-A2 in the domestic cow and thus, when compared with species from other families, can lead to the assumption that the domestic cow is closer to Cervidae species which lack Bov-A2 at this location as well. In the case of Tragelaphini, one of Bovinae’s tribes, two insertion events were evident yet at a different location from the rest of the family, 39 bp downstream to the common Antilopinae insertion site. As will be described shortly, the reason for the different location and double insertion is sequence related and we argue that it is dictated by a single point mutation. Point mutations, the simplest genetic alteration, can thus lead to different retroposon dispersal in very close species and can mislead evolutionary scientists that use these single events as genetic markers for phylogenetic analysis.

The insertion event that took place in the Antilopinae subfamily is found similarly in all of the explored Antilopinae tribes, indicating that this event is very ancient and dates back at least 23MYA, to Antilopinae pre-speciation time [22]. This insertion event in a very important genomic “strategic” point might have had a significant functional value for the first Antilopinae representatives that migrated from Euro-Asia to Africa [23]. The double insertion events in Tragelaphini obviously took place after Bov-A2 inserted to Antilopinae’ TP53P1 promoter, around 14MYA, as Bovini members (the cow/buffalo tribe) lack Bov-A2 in TP53 P1 promoter [43]. In terms of evolutionary significance, it is interesting to note that a Tragelaphini member (the common eland), which according to the fossil record appeared in Africa between 5.5 MYA to 10.5 MYA [43], has two sequential Bov-A2 elements in the TP53 P1 promoter. In that sense, the common eland is more similar to its African relatives and neighbors in the savanna, the Antilopinae subfamily that have one Bov-A2 element, rather than to its own Euro-Asian subfamily relatives that lack it [23]. This might be a case of convergent evolution in which species from different subfamilies share a common trait due to similarity in their ecological niche.

The Bov-A2 element that was found here is mostly very similar to the previously described sequence in the literature [16]. The major differences are highlighted in Figure 3. When looking into these mutational differences and comparing them to the Bov-A2 consensus, one can clearly identify 4 mutations that are unique to Antilopinae (including the Tibetan antelope), 2 mutations that can separate Caprini (including the Tibetan antelope) and the wildebeest from the Antilopinae sequence, and one mutation unique to Caprini and the wildebeest. The sheep and wildebeest have 3 unique mutations, and the addax and gazelle share one unique mutation. Along with the above similarities, each inspected species has its own unique mutations, apart from the ibex and goat which have identical sequences. The Tibetan antelope, an endangered species from the Tibetan Plateau, is gaining scientific and medical attention due to its ability to live in hypoxic conditions and with high levels of ultraviolet radiation [44]. While there is some debate over the phylogenetic relationship of Tibetan antelopes, our data support the notion that the Tibetan antelope is a part of Antilopinae and more closely related to Caprini [45].

The insertion of Bov-A2 in unrelated insertion events in Antilopinae and Tragelaphini was made possible due to short palindromic sequences that can form small stem and loop structures, designated as PALI1 and PALI2. While PALI1, the entrance site for the Antilopinae Bov-A2 element in the TP53 P1 promoter, is highly conserved throughout the subfamily and beyond (Tragelaphini as well as in the outgroups, Giraffidae and Cervidae), in the domestic cow (both European and Indian) a G to A transversion mutation occurred and probably interfered with the ability of this palindromic sequence to form a stem and loop structure (Figure 3 and Additional file 1: Figure S1). This point mutation might be the cause of the absence of Bov-A2 in the extinct founder of the Bovini. As mentioned before, the insertion event in Antilopinae probably took place very early during Bovidae divergence into two subfamilies. The fact that this mutation occurred only in Bovini might have a significant role in this tribe's evolution. In the case of the water buffalo, which also belongs to Bovini, the existence of the full PALI1 is probably due to a later reversal mutation back to the original sequence as it lacks the Bov-A2 element, like its tribal fellows the cows. The entrance site in Tragelaphini is a 16 bp-long palindromic sequence, PALI2. This sequence is unique in the common eland and lengthens a shorter and thus less energetically favorable 14 bp palindrome that exists in all bovid species. It is hard to speculate whether Bov-A2 entered after PALI2, 39 bp downstream to PALI1, because PALI2 is more “transposon favorable” or that as in the case of the water buffalo its original PALI1 sequence was mutated (like in Bovini) and only later reversed to the more functional one. It was reported earlier that palindromic AT rich sequences can be responsible for genome rearrangements [46] and transposon reintegration [47]. Although the former reported sequences are hundreds of nucleotides in length, and the latter is related to the Tol2 transposon, these findings support our proposed model, with PALI1 and PALI2 being the first reported cases of short palindromic sequences responsible for SINE insertion.

Another interesting finding, related to the insertion site of these Bov-A2 elements into the TP53 P1 promoter, is the mechanism of insertion. It is well known that SINE elements need LINE elements to re-transpose and they do so through an unequal crossing over mechanism [48]. In the case of both Bov-A2 insertions reported here (in Bovinae and Tragelaphini), a partial palindromic sequence was replicated and is evident adjacent to the 3′ end of the Bov-A2 element. This partially replicated palindromic sequence was sufficient in the case of the common eland to enable a second Bov-A2 insertion, resulting in two sequential Bov-A2 elements, rather than Bov-A4 [16]. In the case of the addax, we speculate that the partial PALI1 followed by a long poly(A) tail is the result of a second unsuccessful SINE insertion event (probably not Bov-A2 due to the poly(A) sequence) as poly(A)’s are part of known SINE insertion mechanisms into the genome [49]. Putting together the single insertion event in Antilopinae, the dual insertion event in Tragelaphini, and the second insertion attempt in the addax (a member of Antilopinae) all in the TP53 P1 promoter, strengthens the hypothesis that this promoter and its short palindromic sequences are hotspots for insertion of genetic elements, which probably provided an evolutionary advantage due to functional significance.

TFBS analysis revealed unique TFBSs that exist only in the inserted Bov-A2 elements and thus might represent a classic case of modified regulation of gene expression which may lead to modified function. The loss of the conserved STAT3 binding site in the LINE sequence of Antilopinae (apart from the wildebeest and sheep which retained this TFBS) and the gain of this same binding site in the Bov-A2 element in proximity to an NFκB binding site, might be a result of a subfunctionalization-like process. As was mentioned above, in the common eland's TP53 promoter, two Bov-A2 insertion events were obtained, in very close proximity to the Antilopinae's Bov-A2 insertion site. This might be a case of convergent evolution in which African members of Bovidae, along with members that live in mountainous rocky regions northern to Africa (Caprinae), have a similar genomic alteration. It was shown experimentally that deletion of STAT3 or its conditional knockout in mice suppresses epithelial apoptosis and dramatically delays mammary gland involution upon forced weaning in mice [50-52]. Moreover, STAT3 along with NFκB signaling pathways are also responsible for inflammatory signaling and acute phase response that are part of later phases of the normal involution process [53], and were shown to be suppressed by Sim2 which promotes delayed involution [54]. TP53 itself is induced rapidly following weaning of neonates [55]. BALB/c-TP53 null mice displayed delayed involution of the mammary epithelium [56]. TP53 is a physiological regulator of mammary involution that acts to rapidly initiate apoptosis in the secretory epithelium [57]. The combined loss of TP53 and STAT3 in double knockout mice, led to a severe perturbation in the mammary gland involution, with hyper delayed loss of epithelium and reappearance of adipocytes. In the absence of STAT3 alone, the apoptotic program can default to a p53 pathway [58]. Activation of NFκB in mammary epithelium promotes milk loss during mammary development [59]. In light of these and our TFBS analysis findings, one may propose that not only STAT3, NFκB and TP53 are involved in the regulation of involution, but also the interplay between these pathways, as the former two TF (presumably via an OR gate) regulate TP53 transcription during the weaning process.

Involution is a complex physiological mechanism, responsible for milk arrest and mammary remodeling upon weaning. Involution was vastly explored molecularly due to its importance to female breast health and due to its economic importance to the dairy market [53]. Although much of our present knowledge of involution, mainly its cellular and underlying molecular mechanism, is based on work with rodents, similarities to dairy animals and studies in bovine explants further support our model. It was shown that in goats and ewes the process of involution has both a similar timeline as well as parallel stages to what is seen in rodents [60,61]. The situation in cows is different as the incidence of apoptosis during tissue remodeling between lactations appears markedly lower [61]. Mammary involution proceeds with little loss of epithelial cells, and no disengagement of epithelial cells from the basement membrane [62]. Mammary explants from pregnant dairy cows have the capacity to maintain a population of surviving mammary epithelial cells that remain hormone-responsive, are capable of milk protein gene expression, and maintain alveolar architecture without exposure to exogenous macromolecules during culture [63]. This intrinsic capacity was attributed to expression of genes which lead to avoidance of cell death pathways [63]. It was proposed that identification of genes that may contribute to cell survival in bovine mammary explants may constitute a significant financial advantage for the dairy industry, due to improved persistence of lactation [64]. These observations, in light of our results, raise the possibility that the absence of Bov-A2 from the TP53 P1 promoter of Bovini domestic species might be the cause for delayed mammary involution.

It is intriguing to speculate that the African Savanna and steep high cliff niches demand its herbivores to wean rapidly and return to normal morphology as soon as possible. The strong predation stress in the Savanna, and the harsh topography that members of Caprini inhabit as compared to the Euro/Asia Bovid habitat, can be strong stressors that favor a shorter involution period. Altogether this might explain the ability of our domestic cows and buffalos to bear greater persistency of lactation.

## Conclusions

We report the independent insertions of Bov-A2 retroposons in the promoter region of TP53, the guardian of the genome, in Antilopinae and Tragelaphini. Whereas these Bov-A2 insertions differ in the genomic location of insertion site, orientation and number of inserted elements, they share a common feature: the entrance site in both cases employed short palindromes that can form hairpin secondary structures. To the best of our knowledge, this is the first report in which short palindromes serve as hot spots for retroposon insertion into the mammalian genome. TFBS analysis of TP3 promoter in Bovidae revealed that the Bov-A2 elements harbor unique binding sites for transcription factors that might regulate TP53 expression and mammary involution. This in turn may answer the need for rapid mammary involution in Bovidae species of the Savanna/mountainous regions. The absence of Bov-A2 in the TP53 promoter of domestic Bovids might have been the early background for future milk persistency.

## Methods

### Biological samples

Biological samples were taken from the following ruminants: Ibex (*Capra nubiana*, n = 5), Goat (*Capra aegagrus hircus*, n = 5), Sheep (*Ovis aries*, n = 2), Wildebeest (*Connochaetes taurinus*, n = 1), Addax (*Addax nasomaculatus*, n = 1), Gazelle (*Gazelle gazelle*, n = 2), Common Eland (*Taurotragus oryx*, n = 1), Water Buffalo (*Bubalus bubalis*, n = 8), Zebu (*Bos indicus*, n = 4), Cow (*Bos taurus*, n = 6), Red Deer (*Cervus elaphus*, n = 3), Spotted Deer (*Axis axis*, n = 3) and Giraffe (*Giraffa camelopardalis*, n = 2). Blood or hair roots were taken from domestic animals, whereas tissue samples were collected from deceased wild animals from the Tel Aviv Ramat Gan Zoological Center. The ibex tissue samples were a generous gift from the collection of Dr. Gila Kahila Bar-Gal from the Hebrew University of Jerusalem.

### Ethics statement

Biological samples were collected by qualified and authorized personnel from accredited institutions, such as the chief veterinarian of the Tel Aviv Ramat Gan Zoological Center, and competent veterinarian authorities at dairy farms and petting zoos.

The biological samples used in this non-experimental research were collected, packed, stored and treated following required standards and appropriate permissions from all relevant parties. According to the Israeli law, the samples collected from animals comply with institutional and national guidelines. Blood samples were taken by authorized veterinarians, as part of routine health procedures, and small portions were shared. Sharing tissues from abattoirs and dead animals do not require the approval of the Institutional Animal Care and Use Committee.

### DNA extraction, amplification and sequencing

DNA was extracted from tissues, blood or hair using the QIAGEN DNA Isolation kit (QIAGEN, Germany).

PCR reactions were carried out in a total volume of 50 microliters with 10 mM Tris–HCl, pH 8.3, 1.5 mM MgCl_2_, 50 mM KCl, 0.2 mM dNTPs, and 30 nM primers. The PCR conditions were 94°C for 5 min, followed by 35 cycles with annealing temperatures of 58°C (depending on the species) for 0.5 min and 72°C for 0.5 min; the reactions were ended with a final extension step at 72°C for 7 min.

Three sequential primers pairs were used to amplify 1500 bp upstream of the TP53 P1 promoter, each pair designed to amplify 500 bp with overlapping sequences. Primers were designed according to BOVIN ENSBTAT00000001420:

- COW TP53P1F1: 5′ ATTGTAGACAAGGTCTCTGCCC 3′
- COW TP53P1R1: 5′ GGGTCATCTAGCGTCCGACC 3′
- COW TP53P1F2: 5′ GAATAAACAGCGTTCCTAAGCC 3′
- COWTP53P1R2: 5′ GCGCATATGGAGGTATACGTTA 3′
- COW TP53P1F3: 5′ ACTCACTAGGAGAACCAAACCCT 3′
- COW TP53P1R3: 5′ GGGATTTGGGTCCACGTTTCCA 3′

PCR products were Sanger sequenced and analyzed on ABI Sequencer (3100) using GeneScan and Genotyper software (PE Applied Biosystems).

The nucleotide sequences were deposited in GenBank (accession numbers: KM233178-KM233190). The Tibetan antelope (*Pantholops hodgsonii*) sequence (from the database) is AGTT01151016.

### Free energy calculation

Free energies [37] and DNA structures were calculated based on the Mfold Web Server [38].

### Transcription factor binding site analysis

Transcription factor binding site (TFBS) analysis was performed using the Genomatix Genome Analyzer (GGA) MatInspector program [39].

## Additional files

Additional file 1: Figure S1. Nucleic acid folding and free energies were calculated by ‘The *mfold* Web Server’ (http://mfold.rit.albany.edu//?q=mfold/DNA-Folding-Form). Asterisk stand for the existence of alternative structures with lower free energy. Additional file 2: Figure S2. Transcription factor binding site (TFBS) analysis was performed using the Genomatix Genome Analyzer (GGA) program (https://www.genomatix.de/solutions/genomatix-genome-analyzer.html). The findings of putative TBFS for each of the promoter sequence of the different species are detailed in separated tabs.

## Abbreviations

BCL: B-Cell CLL/Lymphoma
GGA: Genomatix Genome Analyzer
LINE: Long interspersed nuclear element
NFκB: Nuclear factor kappa B
SINE: Short interspersed nuclear element
STAT: Signal transducers and activators of transcription
TFBS: Transcription factor binding site
TSS: Transcription start site
ZNF: Zinc finger
